# Overexpression of *MMP13* in human osteoarthritic cartilage is associated with the SMAD-independent TGF-β signalling pathway

**DOI:** 10.1186/s13075-015-0788-x

**Published:** 2015-09-23

**Authors:** Erfan Aref-Eshghi, Ming Liu, Patricia E. Harper, Jules Doré, Glynn Martin, Andrew Furey, Roger Green, Proton Rahman, Guangju Zhai

**Affiliations:** Discipline of Genetics, Faculty of Medicine, Memorial University of Newfoundland, St. John’s, NL A1B 3V6 Canada; Division of Biomedical Science, Faculty of Medicine, Memorial University of Newfoundland, St. John’s, NL A1B 3V6 Canada; Division of Orthopedics, Faculty of Medicine, Memorial University of Newfoundland, St. John’s, NL A1B 3V6 Canada; Discipline of Medicine, Faculty of Medicine, Memorial University of Newfoundland, St. John’s, NL A1B 3V6 Canada; Department of Twin Research & Genetic Epidemiology, King’s College London, London, SE1 7EH UK

## Abstract

**Introduction:**

In vitro and animal model of osteoarthritis (OA) studies suggest that TGF-β signalling is involved in OA, but human data is limited. We undertook this study to elucidate the role of TGF-β signalling pathway in OA by comparing the expression levels of *TGFB1* and *BMP2* as ligands, *SMAD3* as an intracellular mediator, and *MMP13* as a targeted gene between human osteoarthritic and healthy cartilage.

**Methods:**

Human cartilage samples were collected from patients undergoing total hip/knee joint replacement surgery due to primary OA or hip fractures as controls. RNA was extracted from the cartilage tissues. Real-time quantitative PCR was performed to measure gene expression. Mann-Whitney test was utilized to compare the expression levels of *TGFB1*, *BMP2, SMAD3* and *MMP13* in human cartilage between OA cases and controls. Spearman’s rank correlation coefficient (*rho*) was calculated to examine the correlation between the expression levels of the four genes studied and non-parametric regression was used to adjust for covariates.

**Results:**

A total of 32 OA cases (25 hip OA and 7 knee OA) and 21 healthy controls were included. The expression of *TGFB1*, *SMAD3*, and *MMP13* were on average 70 %, 46 %, and 355 % higher, respectively, whereas the expression of *BMP2* was 88 % lower, in OA-affected cartilage than that of controls (all *p* < 0.03), but no difference was observed between hip and knee OA (all *p* > 0.4). The expression of *TGFB1* was correlated with the expression of *SMAD3* (*rho* = 0.50, *p* = 0.003) and *MMP13* (*rho* = 0.46, *p* = 0.007) in OA-affected cartilage and the significance became stronger after adjustment for age, sex, and BMI. The expression of *BMP2* was negatively correlated with both *TGFB1* (*rho* = −0.50, *p* = 0.02) and *MMP13* (*rho* = −0.48, *p* = 0.02) in healthy cartilage, but the significance was altered after adjustment for the covariates. There was no correlation between the expression of *SMAD3* and *MMP13*.

**Conclusions:**

Our results demonstrate that *MMP13* expression is associated with an increased expression of *TGFB1* in OA-affected cartilage, possibly through SMAD-independent TGF-β pathway. Furthermore, TGF-β/SMAD3 is overactivated in OA cartilage; yet, the consequence of this overactivation remains to be established.

## Introduction

Osteoarthritis (OA), the most common rheumatic condition, is primarily a disease of articular cartilage and subchondral bone [1]. It presents with joint pain, stiffness, deformity, and joint failure at advanced stage [2], and imposes a high socio-economic burden on society [3]. Although the pathogenesis of OA remains elusive, mounting evidence suggests that transforming growth factor β (TGF-β) signalling plays a role in the development of OA [4].

TGF-β signalling is involved in diverse cellular processes including proliferation, differentiation, migration and apoptosis, as well as extracellular matrix (ECM) synthesis and degradation [5]. It plays a critical role in the development, homeostasis, and repair of the cartilage [4]. Population-based association studies have identified genetic variants in different components of TGF-β signalling to be associated with OA. A single nucleotide polymorphism (SNP; T29 > C) in the transforming growth factor beta 1 (*TGFB1*) gene was reported to be associated with incidence of spinal osteophyte formation in a Japanese population [6]. SNPs, rs2278422 and rs8179181, located in the 6^th^ intron of *TGFB1*, have been associated with knee and hip OA susceptibility in a British Caucasian population [7]. Camurati–Engelmann disease (CED), which presents with long bone steosclerosis, is caused by mutations in *TGFB1*, which lead to elevated TGF-β1 activity [8].

The asporin gene (*ASPN)* has been shown to inhibit TGF-β signalling-mediated synthesis of cartilage-specific ECM components, such as type II collagen (COL2A1) and aggrecan (AGC1) in chondrocytes [9]. The aspartic acid (D) repeat polymorphism in *ASPN* has been associated with OA in Asian populations [9, 10]. When compared to common *ASPN* D13 allele, the D14 allele was found to be overrepresented in knee and hip OA patients, relative to healthy controls [9], resulting in greater inhibitory effects on TGF-β-induced expression of *AGC1* and *COL2A1* [11]. Additionally, the *ASPN* variant, rs13301537, was recently reported to contribute to knee OA risk in the Chinese Han population [12].

Apart from *TGFB1*, other components of the TGF-β signalling pathways have also reported to be associated with OA. Growth differentiation factor 5 (*GDF5*), a member of the TGF-β superfamily, has been associated with OA in Asian and European populations [12–14]. The risk allele (T) in SNP rs143383 (T/C), located in the promoter of *GDF5*, was found to confer lower *GDF5* transcription activity both in vitro and human cartilage [13, 15]. Mutations in the *GDF5* gene have also been associated with other skeletal disorders such as congenital hip dysplasia, Hunter-Thompson-type acromesomelic dysplasia, type C brachydactyly, and Grebe-type chondrodysplasia [4, 16].

In an attempt to replicate OA-associated loci in the Newfoundland and Labrador population, we reported an association between SNP rs1049007 located in the bone morphogenetic protein 2 (*BMP2*) gene and OA [17]. *BMP2* is also a member of the TGF-β superfamily. Given the SNP is a synonymous polymorphism, what the relationship between the SNP and OA is remains to be discovered. Mutations in mothers against decapentaplegic homolog 3 (*SMAD3*), one of the intracellular mediators of TGF-β signalling, are known to cause the aneurysm-osteoarthritis syndrome, presenting with early-onset polyarticular OA [18]. In our previous study we found a SNP in the last intron of *SMAD3* to be associated with the total burden of radiographic OA [19], although the exact mechanism for the association needs to be established.

However, most of these studies focused on a single gene at a time. Given that those genetic variants are not functional, how these genes exert their effect on OA remains to be investigated. We, therefore, undertook the current study to elucidate the role of TGF-β signalling pathway in OA by simultaneously examining the expression levels and pair-wise correlations of four genes including *TGFB1* and *BMP2* as ligands, *SMAD3* as an intracellular mediator, and matrix metallopeptidase 13 (*MMP13*) as a targeted gene in human cartilage tissues obtained from OA patients and healthy controls.

## Methods

### Subjects

The study was part of the ongoing Newfoundland Osteoarthritis Study (NFOAS) that was initiated in 2011, aiming at identifying novel genetic, epigenetic, and biochemical markers for OA [20]. OA patients were recruited from those who underwent total knee or hip joint replacement due to primary OA between November 2011 and December 2013 in St. Clare’s Mercy Hospital and Health Science Centre General Hospital in St. John’s, the capital city of Newfoundland and Labrador (NL), Canada. Healthy controls were recruited from the same hospitals from those who underwent hemi-arthroplasty of the hip due to fractures of the femoral neck but did not have evidence of hip OA based on their hip X-ray data, which were further confirmed by pathological examination on the removed femoral head cartilage. OA diagnosis was made based on the American College of Rheumatology criteria [21, 22], and the judgement of the attending orthopaedic surgeons. The pathology reports on the removed cartilage were reviewed for all subjects to ensure the accuracy of the diagnosis and the status of any cartilage degeneration in the controls. The consent rate of the study was 90 %. The study was approved by the Health Research Ethics Authority (HREA) of Newfoundland and Labrador and a written consent was obtained from all study participants.

### Demographics and anthropometrics

Demographic information was obtained by a self-administered questionnaire with the help of the research staff, if necessary. Anthropometric data including height and weight was retrieved from their hospital admission and medical records and body mass index (BMI) was calculated by dividing weight in kilograms by squared height in meters. Age was calculated at the time of the surgery.

### RNA isolation

Four pieces (approximately 200 mg each) of cartilage tissues were retained from either the tibial plateau or femoral heads during the surgery. The samples were then flash frozen and stored in liquid nitrogen until the experiment. Up to 200 mg frozen cartilage tissue was transferred to the homogenizing cylinder with 1 ml TRIzol lysis reagent and 200 μl guanidine thiocyanate and homogenized using a cryogenic mill (Spex Freezer Mill, model 6770, Metuchen, NJ, USA) with the following parameters: two cycles of 2 min grinding at maximum frequency with 10 min cooling down between grinding cycles. The homogenate was then transferred to a new 2 ml RNase-free tube and incubated for 5 min at room temperature. RNeasy Lipid Tissue Mini Kit (Qiagen, Venlo, Netherlands) was used for extracting total ribonucleic acid (RNA) from the aqueous phase as per the manufacturers’ protocol.

### Gene expression measurement

Complementary DNA (cDNA) synthesis from the extracted RNA was done using Maxima H Minus First Strand cDNA Synthesis Kit (K1682, Vilnius, Lithuania). One hundred ng of RNA from each sample was primed with random primers before addition of a reverse-transcription solution [5x buffer, Ribolock, 500 μM deoxyribose nucleoside triphosphates (dNTPs) (Life Technologies, Carlsbad, CA, USA) Maxima polymerase]. One μl of synthesized cDNA was subject to quality control by polymerase chain reaction (PCR) amplification of the target genes and glyceraldehyde 3-phosphate dehydrogenase (*GAPDH*) followed by agarose gel electrophoresis.

Expression quantification of the genes studied was performed using ABI 7900HT Fast Real-Time PCR System on 96-well plate. *GAPDH* was used as an internal reference gene to normalize the relative quantification of the targeted genes—*TGFB1*, *BMP2, SMAD3,* and *MMP13.* Amplification primers were designed using The National Center for Biotechnology Information (NCBI) primer-blast tool for the shortest isoforms of the genes and the sequences were blasted in NCBI Basic Local Alignment Search Tool (BLAST) tool to ensure 100 % coverage of all the isoforms, as well as minor similarly to other genomic sequences. Primers were validated using a 4-point dilution series of two random cDNA samples. Table 1 presents the primer sequences used and the size of PCR products. qPCR was then performed in triplicate using SYBR Green (Power SYBR® Green PCR Master Mix, 4367659, Applied Biosystems, Carlsbad, CA, USA), and forward and reverse primers in a final volume of 20 μl. Cycling conditions were: 95 °C for 10 min, 95 °C for 15 sec, and 60 °C for 1 min, repeated in 45 cycles, followed by melt-curve analysis. One of the control samples was selected as calibrator and the relative quantification (RQ) of the target gene expression in each sample was calculated as fold changes in relation to the calibrator using Livak method [23].

**Table 1:** Primers used in qPCR experiments

	Primer sequence (5′ > 3′)	Product size
*SMAD3* reverse primer	GGCTCGCAGTAGGTAACTGG	91 bp
*SMAD3* forward primer	GCATGGACGCAGGTTCTCC	
*TGFB1* reverse primer	CTCAATTTCCCCTCCACGGC	114 bp
*TGFB1* forward primer	TCCTGGCGATACCTCAGCAA	
*MMP13* reverse primer	AGGTAGCGCTCTGCAAACTGG	92 bp
*MMP13* forward primer	AGCTGGACTCATTGTCGGGC	
*BMP2* reverse primer	CTTGCGCCAGGTCCTTTGAC	111 bp
*BMP2* forward primer	CCACCATGGTCGACCTTTAGGA	
*GAPDH* reverse primer	TCGCCCCACTTGATTTTGG	106 bp
*GAPDH* forward primer	GCAAATTCCATGGCACCGT	

*qPCR* quantitative polymerase chain reaction

### Statistics

Descriptive statistics was summarized using either mean or percentage and comparisons between OA cases and controls were performed using Student’s *t* test or chi-square test wherever appropriate. Mann-Whitney test was utilized to compare gene expression levels between OA cases and controls. Spearman’s rank correlation coefficient (*rho*) was calculated to examine the correlation between the expression levels of the four genes studied and non-parametric regression model was used to adjust for potential confounding factors including age, sex, and BMI. The association between each of the genes studied and the covariates including age, sex, and BMI were also examined. All the statistical analysis was conducted using STATA/SE 11.2 (Stata Corp, College Station, TX, USA). Significance level was defined as at α level of 0.05 without making adjustment for multiple comparisons because it is preferable in this case since the data under evaluation are not random numbers but actual observations [24].

## Results

A total of 53 study participants were included in the study: 32 (7 knee OA and 25 hip OA) OA cases and 21 healthy controls. All of them were Caucasians. Seventy percent of the study participants were females and 30 % were males. Controls were on average 12 years older than OA cases (*p* = 0.0002) and had a lower BMI than OA cases (*p* < 0.0001). Table 2 presents the characteristics of the study population.

**Table 2:** Characteristics of the study population^a^

	Controls (*n* = 21)	OA (*n* = 32)	*p*
Age (yrs)	76.45 ± 10.93	64.30 ± 10.43	0.0002
BMI (kg/m^2^)	23.79 ± 1.03	32.25 ± 1.35	<0.0001
Sex (% females)	76 %	66 %	0.4

*OA* osteoarthritis, *BMI* body mass index

^a^Values are expressed as mean ± standard deviation, unless indicated otherwise

### Gene expression differences between OA and controls

Pathological reports on the cartilage confirmed the status of all the OA cases. It also found that 14 controls had healthy intact cartilage, but 7 other controls had age-related minor degenerative changes in their cartilage. We therefore compared the differences in the expression of the four genes between the healthy intact cartilage samples and those with age-related minor degenerative changes in the controls. We found that there was no difference (all *p* > 0.2; Table 3). Then, we compared the expression of the four genes between hip and knee OA cases; again, we found no difference (all *p* > 0.13; Table 3). Consequently, we combined hip OA and knee OA together and compared them with the data from all 21 controls.

**Table 3:** Gene expression comparison between hip vs. knee OA, and intact healthy cartilage vs. cartilage with minor degeneration in controls^a^

Gene	Hip OA	Knee OA	*p*	Healthy controls	Controls with minor degeneration	*p*
	(*n* = 7)	(*n* = 25)		(*n* = 14)	(*n* = 7)	
*TGFB1*	6.40 ± 2.16	5.71 ± 2.51	0.42	3.35 ± 1.74	4.36 ± 1.89	0.20
*SMAD3*	2.55 ± 1.59	2.62 ± 0.76	0.56	1.84 ± 1.46	1.60 ± 1.14	1.00
*MMP13*	1.74 ± 2.21	0.90 ± 0.74	0.53	0.31 ± 0.53	0.41 ± 0.80	0.82
*BMP2*	0.15 ± 0.02	0.08 ± 0.01	0.13	1.17 ± 0.31	1.23 ± 0.46	0.65

*OA* osteoarthritis

^a^Figures are relative quantification (RQ) mean ± standard deviation

**Fig. 1:**
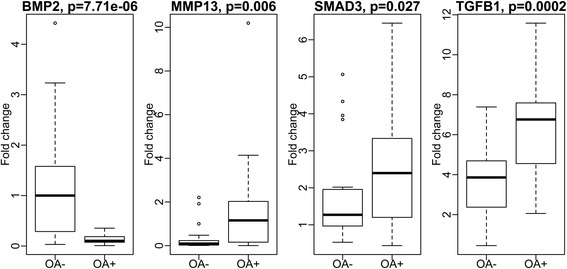
Comparison of the expression levels of *BMP2*, *TGFB1*, *SMAD3*, and *MMP13* in human cartilage between osteoarthritis (OA) cases and controls. *p* values were from Mann-Whitney test

We found that all four genes were expressed in OA-affected and healthy cartilage. The expression of *TGFB1*, *SMAD3* and *MMP13* was on average 70 %, 46 %, and 355 % higher, respectively, whereas the expression of *BMP2* was 88 % lower, in OA-affected cartilage than that in controls (all *p* < 0.03) (Fig. 1).

The expressions of *TGFB1*, *BMP2*, and *MMP13* were not associated with age, sex, and BMI either in OA cases or in controls (all *p* > 0.09). However, we found that the expression of *SMAD3* was correlated with age (*rho* = −0.35, *p* = 0.05) and BMI (*rho* = 0.38, *p* = 0.03). *SMAD3* was also expressed higher in females than in males (48 % higher, *p* = 0.04). These differences were only observed in individuals with OA but not between controls.

### Relationship between *TGFB1*, *BMP2*, *SMAD3*, and *MMP13* expression

We found that the expression of *TGFB1* was significantly correlated with the expression of *SMAD3* (*rho* = 0.50, *p* = 0.003) and *MMP13* (*rho* = 0.46, *p* = 0.007) in OA-affected cartilage but not in healthy cartilage (Table 4). The significance became even stronger after adjustment for age, sex, and BMI (*p* = 0.002 and *p* < 0.0001, respectively).

**Table 4:** Spearman’s correlation coefficients (*rho*) between the expression of *TGFB1*, *BMP2*, *SMAD3*, and *MMP13* in OA-affected and healthy cartilage, respectively

	Controls (*N* = 21)	OA (*N* = 32)
*TGFB1 and SMAD3*	*rho* = 0.07, *p* = 0.7	*rho* = 0.50, *p* = 0.003
*TGFB1 and MMP13*	*rho* = 0.28, *p* = 0.2	*rho* = 0.46, *p* = 0.007
*SMAD3 and MMP13*	*rho* = 0.05, *p* = 0.83	*rho* = 0.15, *p* = 0.39
*BMP2 and TGFB1*	*rho* = −0.50, *p* = 0.02	*rho* = 0.24, *p* = 0.21
*BMP2 and MMP13*	*rho* = −0.48, *p* = 0.03	*rho* = 0.17, *p* = 0.35

*OA* osteoarthritis

We also found that the expression of *BMP2* was negatively correlated with both *TGFB1* (*rho* = −0.50, *p* = 0.02) and *MMP13* (*rho* = −0.48, *p* = 0.02) in healthy cartilage but not in OA-affected cartilage (Table 4). However, the significances were altered after adjustment for age, sex, and BMI.

We found there was no correlation between the expression of *SMAD3* and *MMP13* either in OA-affected cartilage or controls (Table 4).

## Discussion

To the best of our knowledge, this is the first study of using human cartilage samples to demonstrate a significant association between the expression of *TGFB1* and *MMP13*; suggesting TGF-β signalling pathway switches its protective role in normal cartilage observed from in vitro studies [4], to a damaging factor in advanced OA, possibly through SMAD-independent TGF-β pathway.

Evidence from animal models of OA indicates that increased expression of *TGFB1* is involved in OA development. Multiple intra-articular injections of TGF-β in mice joint results in changes to articular cartilage with strong resemblance to both experimental and spontaneous mice OA [25]. High concentrations of active *TGFB1* in the mice subchondral bone is reported to initiate osteoarthritic changes in the bone and cartilage [26], and induced expression of *TGFB1* from the synovial lining layers results in OA-like changes in the murine knee joint including hyperplasia of synovium and chondro-osteophyte formation [27]. Data from human joint tissue, however, are limited. Pombo-Suarez et al. [28] studied cartilage samples obtained from 11 patients with hip OA and 11 patients with femoral neck fracture and found that all three *TGFB* isoforms including *TGFB1* were significantly and highly expressed in osteoarthritic cartilage. Our results are consistent with theirs, demonstrating a 70 % increase in *TGFB1* expression in OA-affected cartilage. Since we only measured mRNA expression of *TGFB1,* our results may not reflect the corresponding protein levels. Pombo-Suarez et al. [28] found that the increased messenger RNA (mRNA) levels of *TGFB* isoforms was in relation to an increased percentage of TGF-β-positive staining chondrocytes, indicating that mRNA expression of *TGFB* isoforms is well correlated to their protein levels. However, Wu et al. [29] performed a proteomic analysis of articular cartilage from 7 knee OA and 7 healthy controls and found a 16-fold decreased protein expression of *TGFB1* in OA cartilage, suggesting the effect of *TGFB1* in OA may be joint specific. We included cartilage samples from both knee and hip OA patients but did not find any difference in the mRNA expression of these three genes. The reason for the discrepancy between our results and Wu’s [29] is unclear. However, possible reasons leading to false positives include sampling bias due to different population sources, control cartilage of unspecified origin, and the utilization of less stringent significance level (raw *p* value < 0.03) given the large number of proteins (*n* = 814) examined in their study. Furthermore, apart from TGF-β1, no other protein involved in the TGF-β signalling was found to be significantly different, indicating caution should be used in interpreting their results.

Verdier et al. [30] reported that expression levels could vary, based on the OA stage and the level of involvement. In the immunohistochemical analysis of cartilage tissues obtained from six hip OA patients and four controls TGF-β1 staining was increased in slightly altered areas, reduced in more degraded cartilage, but markedly increased in the osteophytes, suggesting *TGFB1* may take part in the hypertrophic stage of the OA process. Unfortunately, we do not have cartilage severity data to assess the distribution of *TGFB1* expression in different layers of cartilage.

The consequence of increased *TGFB1* activity is unknown. In vitro studies showed that activity of TGF-β sub-pathway had a protective role in articular cartilage [4]. However, Pombo-Suarez et al. [28] found that none of the expression levels of the three isoforms of *TGF-β* were correlated with the expressions of main proteins in human cartilage, i.e., *COL2A1* and *AGC1*, suggesting the expected role of TGF-β pathway is altered in human OA cartilage. Moldovan et al. [31] found that TGF-β can upregulate the levels of *MMP13* in cultured cartilage explants and cause a mimicking of the in situ distribution of the increased *MMP13* observed in both OA- and rheumatoid arthritis-affected cartilage. Our results are consistent with theirs with a strong correlation between expressions of *TGFB1*and *MMP13* in OA-affected cartilage, suggesting TGF-β switches from a protective role observed from in vitro studies to a damaging factor in OA-affected cartilage. Similar phenomenon has also been reported for other tissues including squamous carcinoma [32], breast cancer [33], human gingival fibroblasts [34] and osteoblasts [35].

*MMP13* is a major enzyme targeting cartilage for the degradation of types II, IV, and IX collagen, proteoglycan, osteonectin and perlecan [36]. Its overexpression has been shown to be related to cartilage destruction among both human OA patients and animal models of OA [37]. It seems that TGF-β signalling regulates expression of *MMP13* through SMAD-dependent pathway in squamous carcinoma cells [32], and in human gingival fibroblasts [34]. In mice primary chondrocytes TGF-β signals through SMAD3 rapidly repress *MMP13* expression, but induce its expression in the absence of SMAD3 [38]. Alternatively, TGF-β has been described as increasing MMP13 expression in osteoblast cells through a combination of SMAD-dependent and SMAD-independent pathways [35]. In the current study we found there was no correlation between *SMAD3* and *MMP13* expression in either normal or OA cartilage, suggesting that the association between *TGFB1* and *MMP13* expression in OA-affected cartilage is primarily through the SMAD-independent pathway.

TGF-β receptors can exert their effect through collateral signalling via mitogen-activated protein kinase (MAPK) and phosphoinositide 3-kinase (PI3K) proteins [39]. The biochemical blockade of MAPK pathway abolishes TGF-β induction of *MMP13* in human breast cancer cell lines [33], and the inhibition of the MAPK pathways reduces *TGFB1*-stimulated *MMP13* expression in the rat osteosarcoma cell line (UMR 106-01) [35], favouring the SMAD-independent pathway for enhanced *MMP13* expression in OA cartilage. The regulation might also occur through other mechanisms including inflammatory factors. It is reported that TGF-β1 treatment increases the expression of pro-inflammatory cytokines, including interleukin-1 (IL-1) and metalloproteinase-1 in synovial fibroblasts from rheumatoid arthritis and normal individuals [40], and IL-1 secretion by chondrocytes has shown to stimulate *MMP13* expression and cartilage degradation in OA [41]. Our data also showed that the increased expression in *MMP13* was disproportional to the increased *TGFB1* expression, suggesting other factors may also play a role in increasing *MMP13* expression in OA cartilage. Blaney Davidson et al. showed that an increase in activin A receptor type II-like 1 (*ALK1*) expression [bone morphogenetic protein (BMP) pathway receptor] was associated with elevated *MMP13* expression in human osteoarthritic cartilage [42], suggesting BMP sub-pathway may also be involved in the regulation of *MMP13* expression in human cartilage. In the current study, we found that *BMP2* expression was negatively associated with both *TGFB1* and *MMP13* expression in healthy cartilage, suggesting that *BMP2* can inhibit *MMP13* expression either directly or indirectly, but this inhibitory effect was disappeared because of the reduced *BMP2* expression in OA-affected cartilage. However, the significant correlation between *BMP2* and *TGFB1/MMP13* was altered after adjustment for potential confounding factors. Sample size might be to blame. We conducted a post hoc power calculation using data on *SMAD3* which had the smallest effect size in our study. For the given sample size and the observed effect size, we had 100 % study power at both α = 0.05 and 0.01. An independent study is needed to confirm the effect of BMP pathway in regulation of *MMP13* expression.

Although *SMAD3* appeared not to be associated with *MMP13* expression in our study, its expression was highly correlated with *TGFB1* and was increased in OA cartilage compared to controls, suggesting that TGF-β/SMAD3 signalling is also overactivated in OA. This enhanced activity may indicate a reparative response by chondrocytes to the cartilage damage resulting from OA progression, through TGF-β/SMAD3 signalling. While TGF-β signals through Smad1/5/8 route are shown to result in deleterious cartilage response, the signals through SMAD2/3 are mainly protective, which indicates that TGF-β/SMAD3 signalling is essential for the cartilage maintenance [43]. In line with this, decreased phosphorylation of SMAD3, an indication of decreased signalling activity, has been reported during OA progression of murine models of OA [44, 45], and *SMAD3* knockout mice have shown to develop OA-like features in their joints [46]. Further studies are needed to elucidate the consequence of the overactivity of TGF- β/SMAD3 pathway.

The strength of the current study is the use of human cartilage rather than animal models or cultured cells, thus having a direct application to OA patients. However, we only studied cartilage tissue, limiting the generalizability of the findings to other joint tissues involved in OA. mRNA expression levels may not reflect the corresponding protein levels, but previous studies found that mRNA levels of *TGFB1* was well correlated with its protein levels [31], suggesting this is not a concern. However, our study is cross-sectional and we cannot conclude a causal relationship.

## Conclusions

We demonstrated that *TGFB1* switches its protective role as observed using in vitro studies to a damaging factor in human OA cartilage, leading to an increased expression of *MMP13*, possibly through SMAD-independent pathway. Further, we found that TGF-β/SMAD3 pathway was also overactivated, but the consequence needs to be established.

## Abbreviations

AGC1: aggrecan
ALK1: activin A receptor type II-like 1
ASPN: asporin gene
BLAST: Basic Local Alignment Search Tool
BMI: body mass index
BMP: bone morphogenetic protein
BMP2: bone morphogenetic protein 2
cDNA: complementary deoxyribonucleic acid
CED: Camurati–Engelmann disease
COL2A1: type II collagen
DNA: deoxyribonucleic acid
dNTP: deoxyribose nucleoside triphosphate
ECM: extracellular matrix
GAPDH: glyceraldehyde 3-phosphate dehydrogenase
GDF5: growth differentiation factor 5
HREA: Health Research Ethics Authority
IL-1: interleukin-1
MAPK: mitogen-activated protein kinase
MMP13: matrix metallopeptidase 13
mRNA: messenger ribonucleic acid
NCBI: The National Center for Biotechnology Information
NFOAS: Newfoundland Osteoarthritis Study
NL: Newfoundland and Labrador
OA: osteoarthritis
PCR: polymerase chain reaction
PI3K: phosphoinositide 3-kinase
*rho*: Spearman’s rank correlation coefficient
RNA: ribonucleic acid
RQ: relative quantification
SMAD3: mothers against decapentaplegic homolog 3
SNP: single nucleotide polymorphism
TGFB1: transforming growth factor beta 1 gene
TGF-β: transforming growth factor beta
